# Managing pasireotide-associated hyperglycemia: a randomized, open-label, Phase IV study

**DOI:** 10.1007/s11102-021-01161-4

**Published:** 2021-07-18

**Authors:** Susan L. Samson, Feng Gu, Ulla Feldt-Rasmussen, Shaoling Zhang, Yerong Yu, Przemysław Witek, Pramila Kalra, Alberto M. Pedroncelli, Philippe Pultar, Nadine Jabbour, Michaela Paul, Marek Bolanowski

**Affiliations:** 1 Baylor College of Medicine, Houston, TX USA; 2 Peking Union Medical College Hospital, Beijing, China; 3 Centre for Cancer and Organ Diseases, Rigshospitalet, Copenhagen University, Copenhagen, Denmark; 4 Sun Yat-Sen Memorial Hospital, Sun Yat-Sen University, Guangzhou, China; 5 West China Hospital, Sichuan University, Chengdu, China; 6 Military Institute of Medicine and Medical University of Warsaw, Warsaw, Poland; 7 MS Ramaiah Medical College and Hospitals, Bengaluru, India; 8 Novartis Pharma AG, Basel, Switzerland; 9 Novartis Pharmaceuticals Corporation, East Hanover, NJ USA; 10 Wroclaw Medical University, Wroclaw, Poland; 11 Present Address: Mayo Clinic, 4500 San Pablo Road, Jacksonville, FL 32224 USA; 12 Present Address: Recordati AG, Basel, Switzerland

**Keywords:** Hyperglycemia, Pasireotide, Insulin, Incretin-based therapy, Acromegaly, Cushing’s

## Abstract

**Purpose:**

Pasireotide is an effective treatment for acromegaly and Cushing’s disease, although treatment-emergent hyperglycemia can occur. The objective of this study was to assess incretin-based therapy versus insulin for managing pasireotide-associated hyperglycemia uncontrolled by metformin/other permitted oral antidiabetic drugs.

**Methods:**

Multicenter, randomized, open-label, Phase IV study comprising a core phase (≤ 16-week pre-randomization period followed by 16-week randomized treatment period) and optional extension (ClinicalTrials.gov ID: NCT02060383). Adults with acromegaly (n = 190) or Cushing’s disease (n = 59) received long-acting (starting 40 mg IM/28 days) or subcutaneous pasireotide (starting 600 µg bid), respectively. Patients with increased fasting plasma glucose (≥ 126 mg/dL on three consecutive days) during the 16-week pre-randomization period despite metformin/other oral antidiabetic drugs were randomized 1:1 to open-label incretin-based therapy (sitagliptin followed by liraglutide) or insulin for another 16 weeks. The primary objective was to evaluate the difference in mean change in HbA_1c_ from randomization to end of core phase between incretin-based therapy and insulin treatment arms.

**Results:**

Eighty-one (32.5%) patients were randomized to incretin-based therapy (n = 38 received sitagliptin, n = 28 subsequently switched to liraglutide; n = 12 received insulin as rescue therapy) or insulin (n = 43). Adjusted mean change in HbA_1c_ between treatment arms was – 0.28% (95% CI – 0.63, 0.08) in favor of incretin-based therapy. The most common AE other than hyperglycemia was diarrhea (incretin-based therapy, 28.9%; insulin, 30.2%). Forty-six (18.5%) patients were managed on metformin (n = 43)/other OAD (n = 3), 103 (41.4%) patients did not require any oral antidiabetic drugs and 19 patients (7.6%) were receiving insulin at baseline and were not randomized.

**Conclusion:**

Many patients receiving pasireotide do not develop hyperglycemia requiring oral antidiabetic drugs. Metformin is an effective initial treatment, followed by incretin-based therapy if needed.

**ClinicalTrials.gov ID**: NCT02060383.

## Introduction

Acromegaly and Cushing’s disease are rare yet highly debilitating endocrine conditions [1, 2]. Acromegaly is most commonly caused by a growth hormone (GH)-secreting pituitary adenoma, whereas Cushing’s disease results from an adrenocorticotropic hormone (ACTH)-secreting pituitary adenoma, with consequent overproduction of insulin-like growth factor 1 (IGF-1) and cortisol, respectively [1]. Chronic hypersecretion of these hormones is associated with significant comorbidities and, if left untreated, increased mortality [3, 4]. Additionally, many patients with Cushing’s disease or acromegaly have underlying impaired glucose tolerance or overt diabetes mellitus because of increased insulin resistance [5, 6].

Pasireotide is a second-generation, multireceptor-targeted somatostatin receptor ligand (SRL) with proven efficacy for the treatment of acromegaly [7, 8] and Cushing’s disease [9, 10]. A subcutaneous (SC) twice-daily formulation of pasireotide is approved for Cushing’s disease [11], whereas a monthly intramuscular (IM) formulation is approved for both acromegaly and, more recently, Cushing’s disease [12]. Pasireotide targets four of the five somatostatin receptor subtypes (SSTRs), with the highest affinity for SSTR_5_, followed by SSTR_2_ [13]. The affinity for SSTR_5_ is several times higher for pasireotide than for octreotide or lanreotide, which explains the increased efficacy of pasireotide for patients with Cushing’s disease or acromegaly [7–9]. By binding these SSTRs, pasireotide reduces secretion of GH and ACTH in patients with acromegaly or Cushing’s disease [14–16]. However, this unique binding profile can also increase blood glucose levels in some patients [7–10], resulting from inhibition of insulin secretion and the incretin response and only modest suppression of glucagon [17, 18], which is reversible upon discontinuation of pasireotide [19]. Nevertheless, pasireotide did not appear to affect insulin sensitivity [17].

Given the unique binding profile of pasireotide and experience from studies in healthy volunteers, incretin-based therapies (dipeptidyl peptidase 4 [DPP-4] inhibitors and glucagon-like peptide 1 [GLP-1] receptor agonists) may be useful for managing hyperglycemia during pasireotide treatment [17, 20, 21]. To our knowledge, this Phase IV trial is the first *prospective* study designed to assess the efficacy of incretin-based therapy versus insulin for the management of pasireotide-associated hyperglycemia that is not fully controlled despite treatment with metformin or other non-incretin-based oral antidiabetic drugs (OADs) in patients with acromegaly or Cushing’s disease.

## Materials and methods

### Patients

The study was conducted in accordance with the Declaration of Helsinki, with an independent ethics committee/institutional review board at each site approving the study protocol. All patients provided written informed consent before participation. Adult patients with confirmed acromegaly (including de novo patients if not surgical candidates) or Cushing’s disease (persistent, recurrent or de novo) were enrolled. Patients receiving pasireotide at screening required fasting plasma glucose (FPG) ≥ 126 mg/dL (≥ 7 mmol/L) on two separate occasions, or a diagnosis of diabetes mellitus (glycated hemoglobin [HbA_1c_] ≥ 6.5% [≥ 48 mmol/mol] or random plasma glucose ≥ 200 mg/dL [≥ 11.0 mmol/L] with classic symptoms of hyperglycemia [polydipsia, polyphagia, polyuria]). Patients receiving OADs other than incretin-based agents were also enrolled. Patients receiving insulin were eligible for study entry but not randomization. Key exclusion criteria were: use of DPP-4 inhibitors or GLP-1 receptor agonists within 4 weeks before study entry; life-threatening diabetic ketoacidosis or diabetic hyperosmolar coma; HbA_1c_ > 10% at screening.

### Study design

**Fig. 1:**
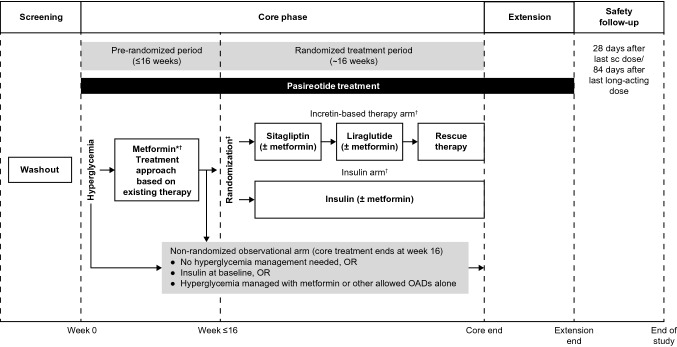
Study design. *Patients initiated metformin upon experiencing SMBG ≥ 126 mg/dL on three consecutive days; patients who could not tolerate metformin or had a contraindication to metformin were randomized immediately; ^†^Patients could continue permitted OADs (other than incretin-based therapies) at the discretion of the investigator; ^‡^Randomization stratified by disease (Cushing’s disease or acromegaly) and baseline glycemic status (HbA_1c_ < 7% or ≥ 7%)

This was a multicenter, randomized, open-label, Phase IV study comprising a core phase (≤ 16-week pre-randomization period followed by 16-week randomized treatment period) and an optional extension phase (Fig. 1). In patients receiving pasireotide at screening, a washout of ≥ 3 months (long acting) or 1 week (twice-daily formulation) was required. For patients receiving treatments other than pasireotide for acromegaly or Cushing’s disease at screening, treatment was discontinued for ≥ 5 times the half-life of the respective formulation before study entry. Cabergoline and pegvisomant were permitted provided the dose regimen was stable for ≥ 4 weeks before study entry and throughout the study. Patients with type 1 or 2 diabetes receiving insulin (basal insulin with/without prandial insulin) were eligible for study entry and treated in the non-randomized observational arm. Patients who received insulin for an acute medical need (subsequently discontinued) required a washout period of ≥ 48 h before study entry; these patients were eligible for randomization. Patients receiving DPP-4 inhibitors or GLP-1 receptor agonists required a ≥ 4-week washout period before study entry.

#### Pre-randomized period

At the start of the core phase, all patients initiated long-acting pasireotide 40 mg IM once/28 days (acromegaly) or pasireotide 600 μg SC twice daily (bid; Cushing’s disease). Dose adjustments were permitted based on biochemical response/tolerability. GH and IGF-1 levels (acromegaly) and urinary free cortisol levels (Cushing’s disease) were assessed at each study site 12 weeks after initiation of long-acting pasireotide and 8 weeks after initiation of pasireotide SC, respectively, and any time thereafter at the investigator’s discretion; data were used by the investigators to guide the titration of pasireotide only and were not recorded in the study database. Dose up-titration of long-acting pasireotide to 60 mg IM once/28 days after 12 weeks was permitted for patients who did not achieve GH < 2.5 µg/L and normal IGF-1. Pasireotide SC could be increased to 900 μg after 8 weeks of treatment.

Dose reduction (decrements of 20 mg IM once/28 days and 300 µg bid) was recommended for patients with self-monitored blood glucose (SMBG) > 300 mg/dL for three consecutive days who were compliant with medication and without comorbidities influencing glucose metabolism. Discontinuation of pasireotide, if SMBG remained > 300 mg/dL for three consecutive days despite optimal antidiabetic therapy, was recommended based on an assessment of benefit and risk by the investigator. Patients naïve to antidiabetic treatment, who experienced increased SMBG (≥ 126 mg/dL on three consecutive days), initiated metformin at a dose of 1000 mg/day bid, adjusted according to approved dosing instructions. Patients on metformin at study entry with elevated SMBG (≥ 126 mg/dL on three consecutive days) while receiving the maximum tolerated and stable dose of metformin were randomized immediately, whereas those receiving submaximal tolerated doses were first titrated to the weekly tolerated dose. Patients on other allowed OADs (non-incretin-based therapies, eg acarbose and sulfonylureas) at study entry, with no contraindication to metformin, received metformin at a starting dose based on the investigator’s judgment, adjusted according to approved dosing instructions; patients could continue other OADs at the discretion of the investigator. Patients who could not tolerate or had a contraindication to metformin continued OAD therapy, with dose modifications permitted according to the investigator’s judgment and approved dosing instructions.

#### Randomized treatment period

Patients with average fasting SMBG ≥ 126 mg/dL on three consecutive days during the 16-week pre-randomization period, despite optimized treatment with metformin/other permitted OADs, were randomized 1:1 to incretin-based therapy (sitagliptin [DPP-4 inhibitor] followed by liraglutide [GLP-1 receptor agonist] rescue therapy) or insulin for another 16 weeks (Fig. 1). Patients who could not tolerate or had a contraindication to metformin were randomized immediately if fasting SMBG was ≥ 126 mg/dL on three consecutive days. Sitagliptin was initiated at 50 or 100 mg/day based on renal function and adjusted according to creatinine clearance in accordance with local prescribing information. Liraglutide and insulin were also administered in accordance with local prescribing information. The suggested starting dose of insulin was 10 IU/day administered at bedtime, with weekly titration to achieve SMBG < 126 mg/dL on three consecutive days. The dose of basal insulin could be down-titrated at any point during the randomized treatment period at the discretion of the investigator. Addition or treatment with prandial insulin (such as insulin regular, lispro, aspart, or glulisine) could be instituted at any time based on investigator discretion to achieve optimal glucose control.

Randomization was stratified by disease (acromegaly, Cushing’s disease) and baseline HbA_1c_ (< 7%, ≥ 7%). A randomization list was produced by an interactive response technology provider using a validated system that automated the random assignment of patient numbers to randomization numbers. Investigators, patients, and the study sponsor were aware of the assigned treatment. Patients randomized to incretin-based therapy were switched from sitagliptin to liraglutide if SMBG was elevated (≥ 126 mg/dL on three consecutive days) on a stable dose of sitagliptin for ≥ 6 weeks (switched earlier if SMBG was > 160 mg/dL). Patients randomized to incretin-based therapy received rescue insulin if HbA_1c_ was centrally confirmed as > 7% or FPG > 160 mg/dL after ≥ 6 weeks of treatment with a stable dose of liraglutide.

#### Non-randomized observational arm

This arm included patients who did not require hyperglycemia management (no OAD group) and those whose hyperglycemia was managed with metformin/other permitted OADs (OAD group). Additionally, patients receiving insulin at study entry were not randomized and were followed up for 16 weeks to evaluate the effect of pasireotide on blood glucose (insulin at entry group). Insulin dose was adjusted based on investigator’s discretion to achieve SMBG < 126 mg/dL and to avoid hypoglycemia.

#### Extension phase

Non-randomized patients who reached the end of the pre-randomization phase, and randomized patients who reached the end of the randomized phase, could continue receiving pasireotide and antidiabetic therapy during an optional extension phase at the investigator’s discretion. Patients continued in the extension until the last patient randomized in the core phase completed 16 weeks of randomized treatment, or until pasireotide was available commercially or through a local access program.

### Objectives and assessments

The primary objective was to evaluate the difference in change in HbA_1c_ from randomization to the end of the core study (16 weeks after randomization) between the incretin-based therapy and insulin arms. Secondary objectives included evaluation and sustainability of glycemic control, as well as safety and tolerability of pasireotide in combination with antidiabetic medication. GH/IGF-1 and urinary free cortisol data were measured locally and used solely for titration of pasireotide and investigator knowledge of disease control; they were not recorded for this study.

HbA_1c_ was measured at a central laboratory at screening, at baseline, once a month throughout the core study (including before starting rescue therapy), and every 8 weeks during the extension. FPG was monitored at the central laboratory every 2 weeks during the core study. After enrollment, patients also self-monitored plasma glucose with a glucometer daily during the core phase, reviewed by the investigator at each visit. During the extension, FPG was monitored every 4 weeks. Adverse events (AEs) and serious AEs (SAEs) were continually assessed and defined using Medical Dictionary for Regulatory Activities v21.0 and graded according to Common Terminology Criteria for Adverse Events (CTCAE) v4.03; relationship to study drug was assessed by the investigator. Patient diabetic status was defined as follows: diabetic, HbA_1c_ ≥ 6.5% and/or FPG ≥ 126 mg/dL at two different visits, prior history of diabetes mellitus, or treatment with antidiabetic medication; pre-diabetic, not qualifying as diabetic and FPG ≥ 100 mg/dL and/or HbA_1c_ 5.7– < 6.5%; normal glucose tolerance, not qualifying as diabetic or pre-diabetic and with FPG < 100 mg/dL and/or HbA_1c_ < 5.7%. Patients classified as diabetic remained in that category for the rest of the study.

### Statistical methods

No formal hypothesis testing was planned. The sample size was calculated to ensure that the half-width of the 95% confidence interval (CI) for the mean difference of the change from randomization to week 16 (the subsequent scheduled visit after 16 weeks of randomized treatment) in HbA_1c_ was approximately 0.5% (assuming a standard deviation of 1.03%). A total sample size of 68 randomized and evaluable patients with at least 8 weeks of randomized treatment without any rescue antidiabetic medication was required. To allow for dropout/rescue rates prior to week 8, approximately 79 patients would be randomized. The total number of enrolled patients was based on the actual randomization rate (which occurred within 16 weeks after patients were enrolled) and was monitored regularly.

The primary variable was change in HbA_1c_ (%) from randomization at 16 weeks to the end of the core phase. For patients who discontinued or required rescue treatment before the time of the primary endpoint assessment, the last HbA_1c_ assessment conducted 8 weeks (56 days) after randomization (and prior to or on the date of start of rescue treatment) was carried forward for the primary efficacy analysis. If the patient discontinued the study or used rescue treatment within 8 weeks after randomization, data were considered missing. An estimate of the mean difference in change from randomization in HbA_1c_ between the two randomized arms was reported with 95% CIs. Variance estimation was based on an analysis of variance (ANOVA) model using the two randomization stratification factors (disease and glycemic status at baseline) and treatment (incretin vs insulin) as fixed effects. As a supportive analysis, the ANOVA for the primary analysis was repeated. Following the intent-to-treat (ITT) principle, the last available HbA_1c_ assessment during the core period in each randomized group was utilized for analyses, regardless of time of discontinuation or rescue treatment used. Secondary endpoints were analyzed descriptively.

## Results

### Study population

**Fig. 2:**
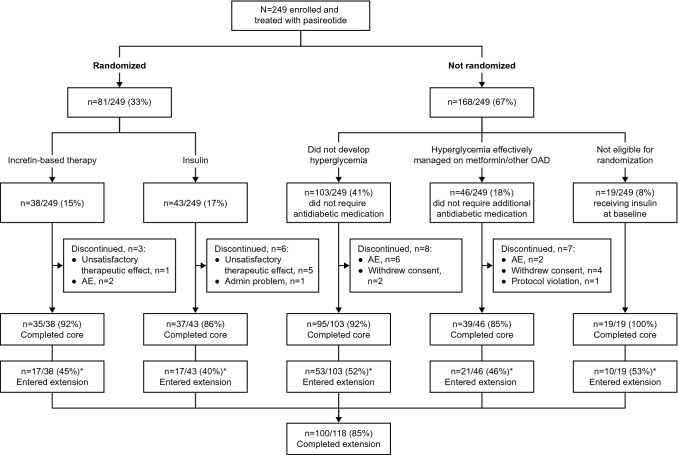
Patient disposition flowchart. *Antidiabetic treatment during the extension phase was based on investigator discretion and may have been different to the treatment received during the core phase

Altogether, 249 patients were enrolled and treated with long-acting pasireotide (n = 190 with acromegaly) or pasireotide SC (n = 59 with Cushing’s disease) between May 2014 and March 2018 (Fig. 2). During the core study, 81 (32.5%) patients required additional antidiabetic therapy and were randomized to incretin-based therapy (n = 38) or insulin (n = 43). Of 168 (67.5%) patients not randomized, 19 were receiving insulin at baseline (insulin at entry observational group), 46 were managed with metformin/other OADs (OAD group), and 103 did not require any antidiabetic medication (no OAD group). The proportion of patients who were receiving insulin at baseline was 6.8% and 10.2% for patients with acromegaly and Cushing’s disease, respectively. For patients with acromegaly, 17.4% were controlled on metformin/other OADs, while for patients with Cushing’s disease, 22.0% were controlled on metformin/other OADs. A greater proportion of patients with acromegaly did not require any antidiabetic medication than those with Cushing’s disease (46.3% vs 25.4%). In total, 175/190 (92.1%) patients with acromegaly and 50/59 (84.7%) with Cushing’s disease completed the core study; 15/190 (7.9%) and 9/59 (15.3%) discontinued, respectively, because of AEs (n = 6 and n = 4), unsatisfactory therapeutic effect of pasireotide (n = 3 and n = 3), withdrawal of consent (n = 5 and n = 1), administrative problems (n = 0 and n = 1) and protocol violation (n = 1 and n = 0).

Median age was 41 years (range 21–79) for patients with acromegaly and 40 years (range 18–72) for patients with Cushing’s disease. Disease history and baseline characteristics in patients with acromegaly or Cushing’s disease were comparable between randomized and non-randomized patients, except for glycemic status in patients with Cushing’s disease, whereby a higher proportion of randomized patients had HbA_1c_ ≥ 7% than non-randomized patients (28.0% vs 8.8%; Tables 1 and 2). At baseline, 34.9% of patients were classified according to study criteria as diabetic (acromegaly: 30.0%; Cushing’s disease: 50.8%), 32.5% were pre-diabetic (acromegaly: 35.3%; Cushing’s disease: 23.7%), and 32.5% had normal glucose tolerance (acromegaly: 34.7%; Cushing’s disease: 25.4%). Of the 81 patients who were randomized, 53 (65.4%) were classified as diabetic, 21 (25.9%) were pre-diabetic, and 7 (8.6%) had normal glucose tolerance at baseline. At the time of randomization, all of these patients were classified as diabetic.

**Table 1:** Acromegaly patient demographics and baseline characteristics, by randomized treatment group

Patients with acromegaly								
	Randomized groups			Non-randomized groups				
	Incretin n = 26	Insulin n = 30	All n = 56	Insulin at entry n = 13	OAD n = 33	No OAD n = 88	All n = 134	All patients n = 190
Median time from diagnosis to first pasireotide dose,^a^ months (range)	44.5 (5.0–244.0)	31.0 (3.0–159.0)	38.5 (3.0–244.0)	29.0 (3.0–255.0)	34.0 (1.0–387.0)	37.5 (0.0–322.0)	36.5 (0.0–387.0)	37.5 (0.0–387.0)
Previous pituitary surgery, n (%)	21 (80.8)	24 (80.0)	45 (80.4)	10 (76.9)	26 (78.8)	82 (93.2)	118 (88.1)	163 (85.8)
Previous medical therapy for acromegaly, n (%)	17 (65.4)	24 (80.0)	41 (73.2)	8 (61.5)	25 (75.8)	69 (78.4)	102 (76.1)	143 (75.3)
Previous pituitary irradiation, n (%)	5 (19.2)	10 (33.3)	15 (26.8)	2 (15.4)	8 (24.2)	29 (33.0)	39 (29.1)	54 (28.4)
Glycemic status, n (%)								
HbA_1c_ < 7%	24 (92.3)	26 (86.7)	50 (89.3)	3 (23.1)	33 (100)	87 (98.9)	123 (91.8)	173 (91.1)
HbA_1c_ ≥ 7%	2 (7.7)	4 (13.3)	6 (10.7)	10 (76.9)	0	0	10 (7.5)	16 (8.4)
Missing	0	0	0	0	0	1 (1.1)	1 (0.7)	1 (0.5)

^a^Time to first pasireotide dose in the study treatment period since diagnosis = (first pasireotide dose date – date of diagnosis + 1) × 12/365.25

**Table 2:** Cushing’s disease patient demographics and baseline characteristics, by randomized treatment group

Patients with Cushing’s disease								
	Randomized patients			Non-randomized patients				
	Incretin n = 12	Insulin n = 13	All n = 25	Insulin at entry n = 6	OAD n = 13	No OAD n = 15	All n = 34	All patients n = 59
Median time from diagnosis to first pasireotide dose,^a^ months (range)	32.0 (7.0–221.0)	40.0 (1.0–332.0)	37.0 (1.0–332.0)	37.5 (14.0–189.0)	35.0 (3.0–163.0)	26.0 (1.0–147.0)	33.0 (1.0–189.0)	33.0 (1.0–332.0)
Cushing’s disease status, n (%)								
De novo	1 (8.3)	2 (15.4)	3 (12.0)	0	2 (15.4)	4 (26.7)	6 (17.6)	9 (15.3)
Persistent/recurrent	11 (91.7)	11 (84.6)	22 (88.0)	6 (100)	11 (84.6)	11 (73.3)	28 (82.4)	50 (84.7)
Previous pituitary surgery, n (%)	11 (91.7)	10 (76.9)	21 (84.0)	6 (100)	13 (100)	11 (73.3)	30 (88.2)	51 (86.4)
Previous medical therapy for Cushing’s disease, n (%)	8 (66.7)	8 (61.5)	16 (64.0)	3 (50.0)	9 (69.2)	10 (66.7)	22 (64.7)	38 (64.4)
Previous pituitary irradiation, n (%)	2 (16.7)	5 (38.5)	7 (28.0)	3 (50.0)	3 (23.1)	4 (26.7)	10 (29.4)	17 (28.8)
Glycemic status, n (%)								
HbA_1c_ < 7%	8 (66.7)	10 (76.9)	18 (72.0)	2 (33.3)	13 (100)	15 (100)	30 (88.2)	48 (81.4)
HbA_1c_ ≥ 7%	4 (33.3)	3 (23.1)	7 (28.0)	3 (50.0)	0	0	3 (8.8)	10 (16.9)
Missing	0	0	0	1 (16.7)	0	0	1 (2.9)	1 (1.7)

^a^Time to first pasireotide dose in the study treatment period since diagnosis = (first pasireotide dose date – date of diagnosis + 1) × 12/365.25

### Treatment exposure

#### Pasireotide

In randomized patients, median (range) duration of exposure to long-acting pasireotide in patients with acromegaly was 5.5 (3.7–7.6) months in the incretin-based therapy arm and 5.5 (4.2–8.0) months in the insulin arm. Patients with Cushing’s disease in the incretin-based therapy and insulin arms received pasireotide SC bid for a median (range) of 4.1 (1.9–6.8) and 4.2 (3.2–5.5) months, respectively.

#### Antidiabetic medications

Of randomized patients who required additional therapy, 79/81 (97.5%) received metformin during the core study, with a similar duration of exposure (Table 3). One patient randomized to insulin erroneously received one dose of sitagliptin prior to randomization, documented as a protocol deviation. All 38 patients randomized to incretin-based therapy received sitagliptin; 28 of them switched to liraglutide (Table 3). Twelve patients (31.6% [Cushing’s disease, n = 6; acromegaly, n = 6]) randomized to incretin-based therapy received insulin as rescue therapy. Of 168 non-randomized patients, 19 were already on insulin, 103 did not require any antidiabetic medication to maintain glycemic control, and 46 were controlled on metformin/OADs (Fig. 2). In the OAD group, 43/46 (95.7%) patients were controlled on metformin alone; three other patients received acarbose (n = 1), gliclazide in combination with linagliptin/metformin (n = 1; discontinued because of protocol deviation), and glibenclamide (n = 1; switched to metformin at baseline visit). For patients who entered the study already on insulin, 15/19 (78.9%) also received metformin (Table 3 and Fig. 2).

**Table 3:** Median duration of exposure to antidiabetic medication during the core phase by treatment group

	Randomized patients			Non-randomized patients				All patients n = 249
	Incretin n = 38	Insulin n = 43	All n = 81	Insulin at entry n = 19	OAD n = 46	No OAD n = 103	All n = 168	
Metformin, n	37	42	79	15	44	0	59	138
Treatment exposure, months (range)	4.6 (2.8–6.9)	4.5 (0.7–7.4)	4.6 (0.7–7.4)	3.7 (2.6–4.0)	2.5 (0.2–3.9)	–	2.8 (0.2–0.4)	3.8 (0.2–7.4)
Sitagliptin, n	38	1	39	0	0	0	0	39
Treatment exposure, months (range)	1.4 (0.1–3.7)	0.03 (0.0–0.0)	1.4 (0.0–3.7)	–	–	–	–	1.4 (0.0–3.7)
Liraglutide, n	28	0	28	0	0	0	0	28
Treatment exposure, months (range)	2.4 (0.2–3.6)	–	2.4 (0.2–3.6)	–	–	–	–	2.4 (0.2–3.6)
Insulin, n	12^a^	43	55	19	0	0	19	74
Treatment exposure, months (range)	1.8 (0.5–3.7)	3.7 (1.4–4.3)	3.7 (0.5–4.3)	3.7 (1.8–3.9)	–	–	3.7 (1.8–3.9)	3.7 (0.5–4.3)

^a^All patients received insulin as rescue therapy

### Glycemic changes in the overall population during the core study

#### Primary efficacy results

At the end of the core phase, estimated difference in adjusted mean change in HbA_1c_ from randomization between incretin-based therapy and insulin (ANOVA, adjusted for randomization stratification factors and treatment) was – 0.28% (95% CI – 0.63, 0.08; Table 4).

**Table 4:** Primary ANOVA comparison of change in HbA_1c_ from randomization until the end of the core phase by randomized treatment arm

	Incretin-based therapy	Insulin
All patients	N = 38	N = 43
Mean, % (95% CI)	– 0.12 (– 0.36, 0.13)	0.26 (– 0.01, 0.53)
Mean difference, % (95% CI)	– 0.28 (– 0.63, 0.08)	
Patients with acromegaly	N = 26	N = 30
Mean, % (95% CI)	– 0.25 (– 0.49, 0.00)	0.19 (– 0.12, 0.49)
Mean difference, % (95% CI)	– 0.36 (– 0.74, 0.02)	
Patients with Cushing’s disease	N = 12	N = 13
Mean, % (95% CI)	0.33 (– 0.41, 1.07)	0.45 (– 0.20, 1.09)
Mean difference, % (95% CI)	– 0.01 (– 0.96, 0.95)	

Nine patients were excluded from the primary analysis because of study discontinuation (n = 2; both with Cushing’s disease, randomized to insulin) or receipt of rescue medication (n = 7 [Cushing’s disease, n = 5; acromegaly, n = 2]) within 8 weeks of randomization. Results of the supportive analysis, based on the ITT principle, were consistent with the primary analysis: adjusted mean change in HbA_1c_ from randomization until end of core phase, 0.0% (95% CI –0.30, 0.30) with incretin-based therapy and 0.24% (95% CI – 0.01, 0.50) with insulin; estimated difference between treatment arms, – 0.24% (95% CI – 0.62, 0.13).

#### Secondary efficacy results

Mean change in HbA_1c_ from randomization until the end of the core phase was 0.0% (95% CI – 0.3, 0.3) with incretin-based therapy and 0.3% (95% CI – 0.0, 0.5) with insulin. Mean change in FPG from randomization until the end of the core phase was –40.1 mg/dL (95% CI – 58.9, – 21.3) and – 36.0 mg/dL (95% CI – 50.8, – 21.2) in the incretin-based therapy and insulin arms, respectively. For non-randomized patients (insulin at entry, OAD, and no OAD subgroups, respectively), mean change in HbA_1c_ from baseline until end of core phase was 1.3% (95% CI 0.6, 1.9), 0.8% (95% CI 0.6, 1.0), and 0.4% (95% CI 0.3, 0.5), and mean change in FPG from baseline until end of core phase was 9.8 mg/dL (95% CI –26.6, 46.3), 22.9 mg/dL (95% CI 15.9, 29.9), and 16.3 mg/dL (95% CI 13.6, 18.9).

Of randomized patients, most with HbA_1c_ < 6.5% at randomization remained in this category at the end of the core phase (all randomized patients: 84.2%; incretin-based therapy: 91.7%; insulin: 71.4%). Up to 20% of patients with HbA_1c_ ≥ 7% at randomization had HbA_1c_ < 7% at the end of the core phase (all: 16.2%; incretin-based therapy: 20%; insulin: 13.6%). Over half of patients with HbA_1c_ 6.5– < 7% at randomization remained in this category or improved to < 6.5% at the end of the core phase (all: 64.0%; incretin-based therapy: 54.5%; insulin: 71.4%). At the end of the core phase, approximately half of all randomized patients had HbA_1c_ < 7% (all: 49.4%; incretin-based therapy: 55.3%; insulin: 44.2%) and 30.9% of patients had HbA_1c_ < 6.5% (incretin-based therapy: 39.5%; insulin: 23.3%; Table 5). In non-randomized patients, overall, HbA_1c_ remained controlled (< 6.5%) in 77.5% of patients with pasireotide (Table 5).

**Table 5:** Shift in HbA_1c_ category from baseline to last core study visit, overall by randomized treatment group

			Last core phase HbA_1c_ value, n (%)			
Patient subgroup	HbA_1c_	Baseline, n (%)	< 6.5%	6.5– < 7%	≥ 7%	Missing
Incretin-based therapy (n = 38)	≥ 7%	15 (39.5)	1 (6.7)	2 (13.3)	12 (80.0)	0
	6.5– < 7%	11 (28.9)	3 (27.3)	3 (27.3)	5 (45.5)	0
	< 6.5%	12 (31.6)	11 (91.7)	1 (8.3)	0	0
	Missing	0	0	0	0	0
	Total	38 (100)	15 (39.5)	6 (15.8)	17 (44.7)	0
Insulin (n = 43)	≥ 7%	22 (51.2)	1 (4.5)	2 (9.1)	19 (86.4)	0
	6.5– < 7%	14 (32.6)	4 (28.6)	6 (42.9)	4 (28.6)	0
	< 6.5%	7 (16.3)	5 (71.4)	1 (14.3)	1 (14.3)	0
	Missing	0	0	0	0	0
	Total	43 (100)	10 (23.3)	9 (20.9)	24 (55.8)	0
All randomized patients (n = 81)	≥ 7%	37 (45.7)	2 (5.4)	4 (10.8)	31 (83.8)	0
	6.5– < 7%	25 (30.9)	7 (28.0)	9 (36.0)	9 (36.0)	0
	< 6.5%	19 (23.5)	16 (84.2)	2 (10.5)	1 (5.3)	0
	Missing	0	0	0	0	0
	Total	81 (100)	25 (30.9)	15 (18.5)	41 (50.6)	0
Insulin at entry (n = 19)	≥ 7%	13 (68.4)	0	0	13 (100)	0
	6.5– < 7%	1 (5.3)	0	0	1 (100)	0
	< 6.5%	5 (26.3)	2 (40.0)	1 (20.0)	2 (40.0)	0
	Missing	0	0	0	0	0
	Total	19 (100)	2 (10.5)	1 (5.3)	16 (84.2)	0
OAD (n = 46)	≥ 7%	0	0	0	0	0
	6.5– < 7%	2 (4.3)	1 (50.0)	0	1 (50.0)	0
	< 6.5%	44 (95.7)	22 (50.0)	14 (31.8)	8 (18.2)	0
	Missing	0	0	0	0	0
	Total	46 (100)	23 (50.0)	14 (30.4)	9 (19.6)	0
No OAD (n = 103)	≥ 7%	0	0	0	0	0
	6.5– < 7%	0	0	0	0	0
	< 6.5%	102 (99.0)	93 (91.2)	8 (7.8)	0	1 (1.0)
	Missing	1 (1.0)	1 (100)	0	0	0
	Total	103 (100)	94 (91.3)	8 (7.8)	0	1 (1.0)
All non-randomized patients (n = 168)	≥ 7%	13 (7.7)	0	0	13 (100)	0
	6.5– < 7%	3 (1.8)	1 (33.3)	0	2 (66.7)	0
	< 6.5%	151 (89.9)	117 (77.5)	23 (15.2)	10 (6.6)	1 (0.7)
	Missing	1 (0.6)	1 (100)	0	0	0
	Total	168 (100)	119 (70.8)	23 (13.7)	25 (14.9)	1 (0.6)

HbA_1c_ category is based on last observed value during the core phase. Baseline refers to randomization to the incretin-based therapy, insulin, and all randomized patients group

### Glycemic changes in patients with acromegaly during the core study

**Fig. 3:**
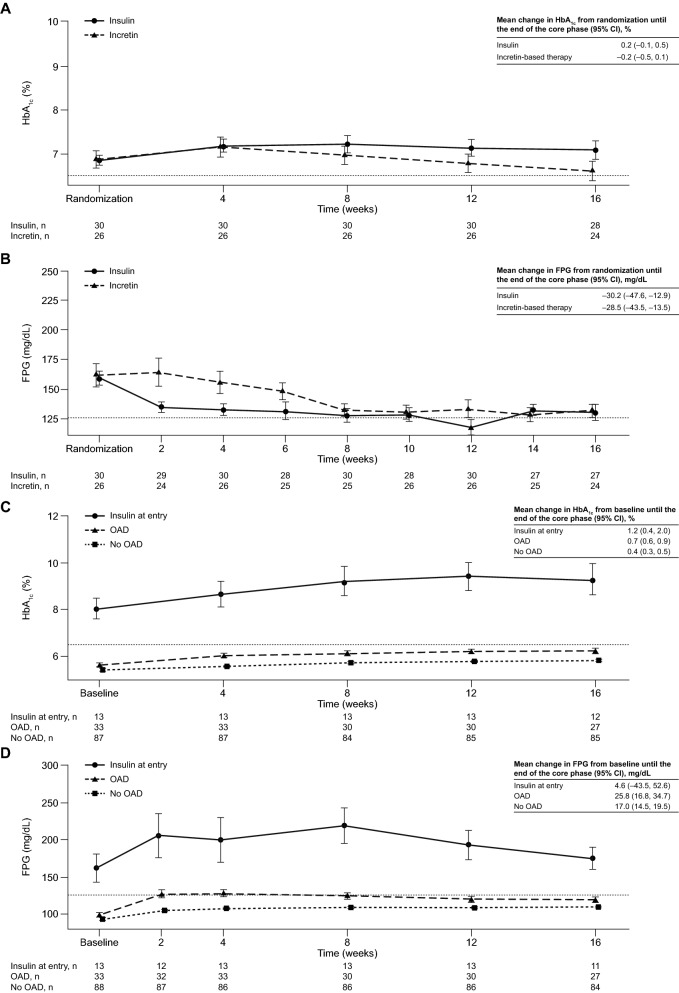
Mean ± SEM **A** HbA_1c_ and **B** FPG levels from randomization to the end of the core study by randomized treatment, and **C** HbA_1c_ and **D** FPG levels from study entry to end of core study by non-randomized treatment, for patients with acromegaly. n refers to the number of patients who contributed to the mean; dashed line is at HbA_1c_ 6.5% in **A** and **C** and at FPG 126 mg/dL in **B** and **D**. SEM, standard error of the mean

In 56 patients randomized with acromegaly, estimated difference in adjusted mean change in HbA_1c_ between the two randomized arms was – 0.36% (95% CI – 0.74, 0.02; Table 4). There was an initial, transient increase in HbA_1c_ from randomization to week 4 in patients treated with incretin-based and insulin therapies (reflecting insufficient glycemic control during the previous 1‒2 months while receiving metformin and/or other OAD medications), followed by a gradual decrease over time. With incretin-based therapy, there was a subsequent gradual decrease in mean HbA_1c_ (Fig. 3A). A reduction in mean FPG values from randomization until the end of the core phase was observed in both treatment groups (Fig. 3B).

Acromegaly patients in the non-randomized no OAD and OAD groups maintained mean HbA_1c_ < 6.5% and FPG < 126 mg/dL with pasireotide treatment, whereas in the insulin at entry group, mean HbA_1c_ and FPG levels were elevated at baseline and remained > 6.5% and > 126 mg/dL, respectively, over time (Fig. 3C and D).

### Glycemic changes in patients with Cushing’s disease during the core study

**Fig. 4:**
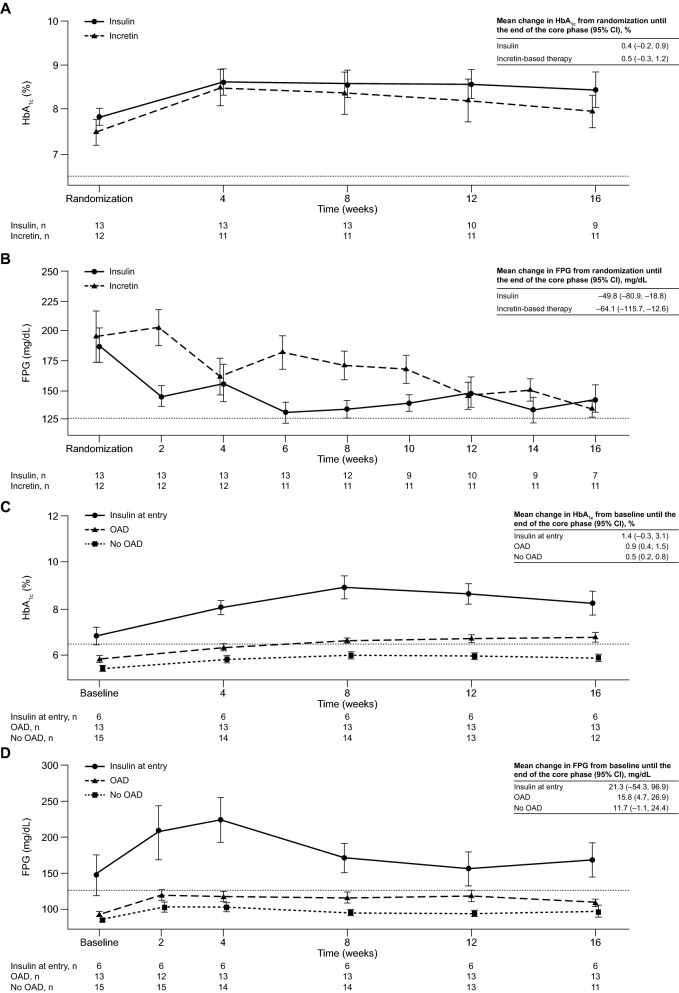
Mean ± SEM **A** HbA_1c_ and **B** FPG levels from randomization to the end of the core study by randomized treatment, and **C** HbA_1c_ and **D** FPG levels from study entry to end of core study by non-randomized treatment, for patients with Cushing’s disease. n refers to the number of patients who contributed to the mean; dashed line is at HbA_1c_ 6.5% in **A** and **C** and at FPG 126 mg/dL in **B** and **D**

In 25 randomized patients with Cushing’s disease, the estimated difference in adjusted mean change in HbA_1c_ between the two randomized arms was – 0.01 (95% CI 0.96, 0.95; Table 4). General trends in mean change in HbA_1c_ and FPG from randomization over time until the end of the core phase were generally similar to those observed in patients with acromegaly, although baseline HbA_1c_ and FPG levels were higher in Cushing’s disease patients at randomization (Fig. 4A and B). Mean changes in HbA_1c_ and FPG for non-randomized patients were similar to those observed in acromegaly patients (Fig. 4C and D).

### Safety

Although hyperglycemia was also recorded as an AE according to CTCAE, it is not discussed here further to the details provided in the previous section. Most patients (92.4%; n = 230/249) experienced one or more AEs, regardless of causality, during the core and/or extension phase, most commonly (≥ 10% of all patients) diarrhea, nausea, and diabetes mellitus (Tables 6 and 7). SAEs of any grade occurred in 22 (8.8%) patients (Table 6). Nine SAEs were suspected to be related to study drug, occurring in no more than one patient each. In total, 43 (17.3%) patients required a reduction in pasireotide dose during the study because of AEs.

**Table 6:** Overview of AEs during the overall study period, by randomized and non-randomized treatment groups

AEs	Randomized patients						Non-randomized patients								All patients n = 249	
	Incretin n = 38		Insulin n = 43		All n = 81		Insulin at entry n = 19		OAD n = 46		No OAD n = 103		All n = 168			
	All, n (%)	Grade ≥ 3, n (%)	All, n (%)	Grade ≥ 3, n (%)	All, n (%)	Grade ≥ 3, n (%)	All, n (%)	Grade ≥ 3, n (%)	All, n (%)	Grade ≥ 3, n (%)	All, n (%)	Grade ≥ 3, n (%)	All, n (%)	Grade ≥ 3, n (%)	All, n (%)	Grade ≥ 3, n (%)
Any AE	37 (97.4)	18 (47.4)	41 (95.3)	8 (18.6)	78 (96.3)	26 (32.1)	18 (94.7)	10 (52.6)	40 (87.0)	3 (6.5)	94 (91.3)	12 (11.7)	152 (90.5)	25 (14.9)	230 (92.4)	51 (20.5)
Drug related	32 (84.2)	8 (21.1)	35 (81.4)	5 (11.6)	67 (82.7)	13 (16.0)	9 (47.4)	5 (26.3)	33 (71.7)	2 (4.3)	66 (64.1)	4 (3.9)	108 (64.3)	11 (6.5)	175 (70.3)	24 (9.6)
Any SAE	6 (15.8)	4 (10.5)	3 (7.0)	3 (7.0)	9 (11.1)	7 (8.6)	4 (21.1)	3 (15.8)	2 (4.3)	0	7 (6.8)	6 (5.8)	13 (7.7)	9 (5.4)	22 (8.8)	16 (6.4)
Drug related	2 (5.3)	1 (2.6)	2 (4.7)	2 (4.7)	4 (4.9)	3 (3.7)	3 (15.8)	2 (10.5)	0	0	2 (1.9)	1 (1.0)	5 (3.0)	3 (1.8)	9 (3.6)	6 (2.4)
AE leading to discontinuation	4 (10.5)	3 (7.9)	1 (2.3)	1 (2.3)	5 (6.2)	4 (4.9)	2 (10.5)	1 (5.3)	2 (4.3)	0	8 (7.8)	5 (4.9)	12 (7.1)	6 (3.6)	17 (6.8)	10 (4.0)
Drug related	2 (5.3)	1 (2.6)	1 (2.3)	1 (2.3)	3 (3.7)	2 (2.5)	1 (5.3)	1 (5.3)	2 (4.3)	0	5 (4.9)	2 (1.9)	8 (4.8)	3 (1.8)	11 (4.4)	5 (2.0)

**Table 7:** Most common AEs (occurring in ≥ 10% of all patients) during the overall study period, by randomized and non-randomized treatment groups

AE	Randomized patients						Non-randomized patients								All patients n = 249	
	Incretin n = 38		Insulin n = 43		All n = 81		Insulin at entry n = 19		OAD n = 46		No OAD n = 103		All n = 168			
	All, n (%)	Grade ≥ 3, n (%)	All, n (%)	Grade ≥ 3, n (%)	All, n (%)	Grade ≥ 3, n (%)	All, n (%)	Grade ≥ 3, n (%)	All, n (%)	Grade ≥ 3, n (%)	All, n (%)	Grade ≥ 3, n (%)	All, n (%)	Grade ≥ 3, n (%)	All, n (%)	Grade ≥ 3, n (%)
Hyperglycemia	15 (39.5)	4 (10.5)	12 (27.9)	3 (7.0)	27 (33.3)	7 (8.6)	7 (36.8)	7 (36.8)	9 (19.6)	0	13 (12.6)	0	29 (17.3)	7 (4.2)	56 (22.5)	14 (5.6)
Diarrhea	11 (28.9)	1 (2.6)	13 (30.2)	0	24 (29.6)	1 (1.2)	2 (10.5)	0	10 (21.7)	1 (2.2)	21 (20.4)	1 (1.0)	33 (19.6)	2 (1.2)	57 (22.9)	3 (1.2)
Nausea	13 (34.2)	1 (2.6)	7 (16.3)	0	20 (24.7)	1 (1.2)	0	0	5 (10.9)	0	11 (10.7)	0	16 (9.5)	0	36 (14.5)	1 (0.4)
Diabetes mellitus	5 (13.2)	1 (2.6)	9 (20.9)	0	14 (17.3)	1 (1.2)	2 (10.5)	1 (5.3)	14 (30.4)	0	4 (3.9)	1 (1.0)	20 (11.9)	2 (1.2)	34 (13.7)	3 (1.2)
Hypoglycemia	6 (15.8)	1 (2.6)	10 (23.3)	0	16 (19.8)	1 (1.2)	8 (42.1)	1 (5.3)	5 (10.9)	0	4 (3.9)	0	17 (10.1)	1 (0.6)	33 (13.3)	2 (0.8)
Upper respiratory tract infection	3 (7.9)	0	3 (7.0)	0	6 (7.4)	0	3 (15.8)	0	6 (13.0)	0	15 (14.6)	0	24 (14.3)	0	30 (12.0)	0
Cholelithiasis	5 (13.2)	0	8 (18.6)	0	13 (16.0)	0	0	0	4 (8.7)	0	9 (8.7)	0	13 (7.7)	0	26 (10.4)	0
Nasopharyngitis	3 (7.9)	0	4 (9.3)	0	7 (8.6)	0	0	0	3 (6.5)	0	16 (15.5)	0	19 (11.3)	0	26 (10.4)	0

During the overall study period, of randomized patients, the most frequently reported AEs of special interest (AESIs; > 10% overall) were hyperglycemia related (54.3%), gallbladder or biliary related (18.5%), and liver safety related (11.1%). Patients in the incretin-based therapy group had a slightly higher incidence of hyperglycemia-related AEs than the insulin group (57.9% vs 51.2%). Patients in the insulin group had a higher incidence of gallbladder- or biliary-related AEs than the incretin-based therapy group (23.3% vs 13.2%). All other AESIs did not appear to be meaningfully different between randomized treatment arms. For non-randomized patients, the most frequently reported AESIs (> 10% overall) were hyperglycemia related (45.2%).

AEs leading to discontinuation occurred in 3.2% and 6.8% of patients with acromegaly and Cushing’s disease, respectively. Four patients randomized to incretin-based therapy discontinued because of AEs: cholecystitis acute, HbA_1c_ increased, urinary tract infection with tubular breast carcinoma during the randomized treatment period, and infectious pleural effusion during the extension phase. One patient randomized to insulin discontinued because of coronary artery stenosis during the extension phase. Two patients in the insulin at entry group (gingival hypertrophy; hyperglycemia), two in the OAD group (arthralgia and myalgia; nausea and vomiting), and eight in the no OAD group (neutropenia; nausea; abdominal pain; pituitary tumor benign; diarrhea; alopecia; subdural hematoma; pregnancy and spontaneous abortion) discontinued because of AEs.

During the randomized treatment period, AEs were reported in 71 (87.7%) patients: 36 (94.7%) receiving incretin-based therapy and 35 (81.4%) receiving insulin, most commonly (≥ 10% of patients overall) hypoglycemia (n = 13; 16.0%), cholelithiasis (n = 12; 14.8%), decreased weight (n = 12; 14.8%), diarrhea (n = 10; 12.3%) and nausea (n = 9; 11.1%). Hypoglycemia and cholelithiasis were more common in patients randomized to insulin than to incretin-based therapy (20.9% vs 10.5% and 16.3% vs 13.2%, respectively), whereas a greater proportion of patients in the incretin-based therapy group experienced decreased weight (23.7% vs 7.0%) and nausea (18.4% vs 4.7%) than in the insulin group.

Two deaths occurred during the study, both during the extension. Neither were suspected to be related to pasireotide; one patient with Cushing’s disease in the insulin at entry group died from febrile neutropenia, and one acromegaly patient in the no OAD group died from a subdural hematoma.

## Discussion

This was the first prospective study to investigate the management of pasireotide-associated hyperglycemia. It was a multicenter study including 249 patients with acromegaly or Cushing’s disease who were treated with pasireotide long-acting release or subcutaneous formulations, respectively. Diabetes is a frequent complication of acromegaly and Cushing’s disease caused by insulin resistance and impaired insulin secretion as a result of excess GH/IGF-1 and cortisol production, respectively [3, 4]. If not contraindicated, metformin is the preferred drug treatment for hyperglycemia [22]. However, the optimal treatment approach for the management of pasireotide-associated hyperglycemia has not been established. Clinical evidence from a healthy volunteer study and expert consensus suggest that hyperglycemia during pasireotide treatment can initially be managed with metformin [20, 21, 23]. Nevertheless, because pasireotide reduces insulin secretion and incretin hormone responses [17], incretin-based therapy may also be an effective option, rather than resorting to insulin initiation by default [20]. This is the first prospective trial to examine this possibility.

Of 81 patients who were randomized to incretin-based therapy or insulin, there was a trend for better control of HbA_1c_ with incretin-based therapy, as indicated in both the primary and supportive analyses. There were also fewer hypoglycemia-related AEs with incretin-based therapy. Overall, two-thirds of patients (168/249; 67.5%) did not meet the criteria for randomization during the 16-week pre-randomization period; 103 did not require antidiabetic medication, 46 were controlled on OADs alone (the majority of whom received metformin alone [93.5%]) and 19 were on insulin from the beginning of the study and did not qualify for randomization. In total, 49.7% of patients with acromegaly and 25.4% with Cushing’s disease did not develop hyperglycemia requiring antidiabetic treatment during the core study, which is in line with safety findings from previous Phase III studies that have shown that many patients do not require antidiabetic therapy during pasireotide treatment [7–10].

Of 81 randomized patients, a trend of increased HbA_1c_ was observed from randomization to week 4 for the overall population (irrespective of underlying disease and treatment group), despite the addition of incretin-based or insulin therapy. As HbA_1c_ is a measurement of average blood glucose over the previous 8–12 weeks [25], the initial increase likely reflected insufficient glycemic control during the 1–2 months prior to randomization. For patients randomized to insulin, initial increases in HbA_1c_ could also be explained by delays in dose titration. This initial rise in HbA_1c_ was followed by a gradual decrease over the remainder of the core study. By core phase end, patients receiving incretin-based therapy generally had a greater decrease in HbA_1c_ than those receiving insulin. A general reduction in mean FPG was also seen in most patients by core phase end, although there was a slight delay in response over the first 8 weeks in patients randomized to incretin-based therapy. This observation might have been caused by initial treatment with sitagliptin; DPP-4 inhibitors augment endogenous incretin action, so the dampened incretin secretion due to pasireotide hampers the efficacy of DPP-4 inhibitors as a glucose-lowering agent [26].

Mean HbA_1c_ levels largely remained controlled (≤ 6.5%) with pasireotide in the no OAD and OAD groups of non-randomized patients with either acromegaly or Cushing’s disease. Despite a small increase in FPG observed in the OAD and no OAD groups, mean FPG remained < 126 mg/dL. Although patients in the insulin at entry group were less controlled (mean HbA_1c_ at core phase end > 7%), most started the study with HbA_1c_ ≥ 7%. Overall, these findings support current medical expert recommendations that metformin should be administered as first-line medical treatment for hyperglycemia during pasireotide therapy, with incretin-based therapies representing an effective option in patients whose hyperglycemia persists [23, 24]. Blood glucose should be monitored in all patients and action taken if necessary; indeed, in our study, almost one-third of patients who were randomized to incretin-based therapy later received insulin rescue therapy. Furthermore, the identification of predictors of response to metformin and other antidiabetic medications would also be of considerable value and would help to guide individualized management of pasireotide-associated hyperglycemia.

AEs observed during the core and/or extension phase of this study were consistent with the known safety profiles of pasireotide and antidiabetic agents [7–10, 20]. Most AEs were of mild-to-moderate severity, and few led to treatment discontinuation. Hyperglycemia-related AEs were generally manageable, with one patient discontinuation as a result. Blood glucose should be monitored in all patients receiving pasireotide, and appropriate action should be taken if levels increase (eg addition of antidiabetic medication) and treatment discontinuation considered if hyperglycemia cannot be properly managed. Incretin-based therapies are known to be associated with gastrointestinal events, consistent with reports of decreased weight, dehydration and hypokalemia [20]. Conversely, a higher proportion of patients in the insulin group reported hypoglycemia, which may be attributed to greater fluctuations in glycemic control with insulin than with incretin-based therapy.

This study is limited by the descriptive nature of our analyses. Furthermore, analysis of the primary objective (change in mean HbA_1c_ levels following 16 weeks of randomized treatment) may have been influenced by the fact that mean change in HbA_1c_ was calculated from the *time of randomization*, at which point mean HbA_1c_ levels had not yet reached peak values. Despite this, the findings from our study suggest a trend that HbA_1c_ is better controlled with incretin-based therapy versus insulin, particularly in patients with acromegaly. GH/IGF-1 and urinary free cortisol data were used to guide therapeutic decisions locally but were not recorded as part of the study design; therefore, we cannot comment on the impact of disease control on the need for antidiabetic therapy. The study protocol also mandated initial treatment with a DPP­4 inhibitor followed by a GLP-1 agonist; therefore, assessing the optimal order of incretin-based therapies was not possible. Decreased secretion of GLP-1 due to pasireotide [17] might indicate a GLP­1 agonist as a rational first choice, as DPP-4 inhibitors can only augment the action of endogenous GLP-1. Overall, 28/38 patients switched to liraglutide. Finally, most (76.3%) patients who participated in this study had acromegaly, which would have influenced the results reported for the analysis of all enrolled patients. Given the greater prevalence of diabetes mellitus and pasireotide-associated hyperglycemia in patients with Cushing’s disease than in those with acromegaly, further investigation of the management of pasireotide-associated hyperglycemia in a larger subset of patients with Cushing’s disease would be valuable.

In summary, these study results highlight that many patients who receive pasireotide treatment do not develop hyperglycemia requiring antidiabetic treatment. For patients who do develop hyperglycemia, metformin alone or in combination with other OAD medications was an appropriate first-line treatment option. When blood glucose was not well controlled, incretin-based therapy was an effective choice for stabilizing HbA_1c_ levels, and insulin was not always required. Overall, 38 patients were randomized to receive sitagliptin, and 28 subsequently switched to liraglutide; 12 received insulin as rescue therapy. There is also the potential for fewer hypoglycemia AEs with incretin-based therapy. Taken together, these findings highlight that hyperglycemia observed during pasireotide treatment is manageable in most patients, without the need for treatment discontinuation.

## Data Availability

Novartis is committed to sharing with qualified external researchers access to patient-level data and supporting clinical documents from eligible studies. These requests are reviewed and approved by an independent review panel on the basis of scientific merit. All data provided are anonymized to respect the privacy of patients who have participated in the trial, in line with applicable laws and regulations. This trial data availability is in accordance with the criteria and process described at www.clinicalstudydatarequest.com.
